# Comprehensive Evaluation of Changes in Placentomes in the Second and Third Trimesters of Pregnancy in Cross‐Bred Hamdani Sheep

**DOI:** 10.1002/vms3.70208

**Published:** 2025-01-24

**Authors:** Banu Kandil, Ali Osman Turgut, Davut Koca, Fatma Isbilir, Muhammed Zahid Atli, Barıs Can Guzel

**Affiliations:** ^1^ Department of Histology and Embryology Faculty of Veterinary Medicine Siirt University Siirt Turkey; ^2^ Department of Animal Science Faculty of Veterinary Medicine Siirt University Siirt Turkey; ^3^ Department of Obstetrics and Gynecology Faculty of Veterinary Medicine Van Yuzuncu Yil University Van Turkey; ^4^ Department of Anatomy Faculty of Veterinary Medicine Siirt University Siirt Turkey

**Keywords:** angiogenesis, histometry, morphometry, placentome, sheep

## Abstract

**Background:**

A proper placentation is required for establishment and continuity of pregnancy. In sheep, placentomes are unique structures that enable nutrition and gas exchange between the mother and the foetus. Although placentomes are dynamic formations, there is limited knowledge of changes in placentomes during pregnancy.

**Objective:**

This study aimed to identify changes in sheep placentomes in the second and third trimesters of pregnancy using both macroscopic and microscopic methods.

**Methods:**

This study investigated 14 healthy cross‐breed Hamdani sheep placentomes, comprising seven second and seven third trimesters of pregnancy. The histomorphometric analysis included measurements of capillary number and area in cotyledonary and caruncular regions, while morphometric assessments encompassed placentome dimensions such as number, length, width, and depth.

**Results:**

Placentomes were oval and circular in shape in the second and third trimesters. In the second trimester, they were observed as concave structures with thick edges, whereas in the third trimester, they were determined as thin‐edged structures with a slight depression in the centre. In the third trimester, foetal and maternal tissues became more intertwined with increased branching of foetal villi and maternal crypts. Placental hematomas and erythrocytes in the cytoplasm of trophoblast cells were more prominent in the third trimester. Statistical analysis revealed no difference in placentome number between the second and third trimesters. However, the dimensions (length, width, and depth) of placentomes were greater in the third trimester compared to the second trimester (*p* < 0.001). Additionally, while there was no difference in the number of cotyledonary versus caruncular capillaries in the second trimester, cotyledonary capillaries outnumbered caruncular capillaries in the third trimester (*p* < 0.001). Furthermore, both cotyledonary and caruncular capillary areas increased in the third trimester compared to the second trimester, with the caruncular capillary area being consistently higher than the cotyledonary capillary area in both trimesters (*p* < 0.05).

**Conclusion:**

This study underscores the substantial structural and physiological transformations of placentomes in the second and third trimesters of pregnancy in sheep. These adaptations facilitate efficient flow exchange between the foetus and mother, highlighting the dynamic nature of placental development during late gestation.

## Graphical abstract

Placentome size, width, and depth increased in the third trimester compared to the second trimester of pregnancy. The caruncular capillary area increased without any significant increase in the number of caruncular capillary vessels in the third trimester compared to the second trimester of pregnancy. On the other hand, both the cotyledonary capillary area and number increased with the progression of the pregnancy.

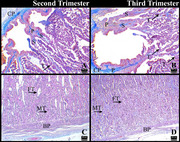

## 1 Introduction

Sheep are among the most significant livestock species, primarily valued for their milk, meat, and wool (Koca et al. 2023; Prache et al. 2022; Scobie et al. 2015; Turgut et al. 2023, 2024). The sustainability of sheep production hinges largely on fertility in breeding programs. Various genetic and environmental factors, including genetic structure, management practices, and diseases, play crucial roles in influencing fertility outcomes in sheep (Ali et al. 2019; Santolaria et al. 2011; Turgut and Koca 2024).

A healthy pregnancy is essential for optimal fertility performance in sheep. Throughout the gestation period of ruminant species, several critical stages, including placentation, are pivotal (Bazer et al. 2009). Proper placentation ensures a healthy connection between the mother and the developing foetus. In sheep, placentation initiates early in pregnancy with the formation of placentomes, which result from the superficial interaction between maternal caruncles and foetal cotyledons (Bazer et al. 2009; Schafer‐Somi 2003). The placentome is a unique structure specific to ruminant species, although its number and distribution vary among different ruminants (Carter and Enders 2013; Chavette Palmer and Tarade 2016). The primary function of the placentome is to facilitate the exchange of nutrients and metabolites between the foetus and the mother (Carter and Enders 2016). This exchange is crucial for supporting the growth and development of the foetus throughout gestation in sheep and other ruminants (Borowicz et al. 2007; Diaz et al. 2015; Reynolds et al. 2005).

A well‐developed vascular network within the placentome is essential for the establishment and maintenance of placentation. During pregnancy, the formation of new blood vessels is achieved through two main processes: vasculogenesis and angiogenesis. Vasculogenesis refers to the formation of blood vessels from precursor cells, while angiogenesis involves the formation of new vessels from pre‐existing vessels (Demir, Yaba, and Huppertz 2010). Angiogenesis is particularly important for the support of healthy pregnancy (Daikoku et al. 2003; Turgut and Korkmaz Ağaoğlu 2023). In ruminants, foetal growth and development are relatively slow until mid‐term pregnancy. However, during the third trimester, foetal development accelerates significantly, largely due to the increased angiogenesis occurring in pregnancy‐related tissues (Diaz et al. 2015). Angiogenesis in these tissues is stimulated by specific angiogenic factors throughout pregnancy (Ağaoğlu et al. 2023; Schafer‐Somi 2003; Turgut et al. 2023). Therefore, angiogenesis plays a critical role in the formation of a healthy placenta and successful pregnancy outcomes in ruminants. It supports the development of a robust vascular network within the placentome, which is essential for effective nutrient and gas exchange between the mother and the foetus throughout pregnancy (Borowicz et al. 2007; Diaz et al. 2015; Reynolds et al. 2005; Turgut and Korkmaz Ağaoğlu 2023).

Placentomes are concave in sheep similar to goats. The number, type, and shape of the placentome may be affected by breed (Vonnahme et al. 2020). The formation and function of the placentome are well studied in sheep (Boshier 1969; Gazali et al. 2023; Majeed, Shalal, and Mohammed 2012; Kashoma and Luziga 2019; Lawn, Chiquoine, and Amoroso 1969). However, limited studies demonstrate morphological and histological structural changes in sheep placentomes during pregnancy (Borowicz et al. 2007; Gazali et al. 2023; Kashoma and Luziga 2019). Considering this background, this study aimed to (i) determine the histological, and histometric changes and (ii) reveal macroscopic and morphometric changes in the placentomes in the second and third trimesters of pregnancy in cross‐bred Hamdani sheep.

## 2 Materials and Methods

### 2.1 Animals and Sampling

**FIGURE 1:**
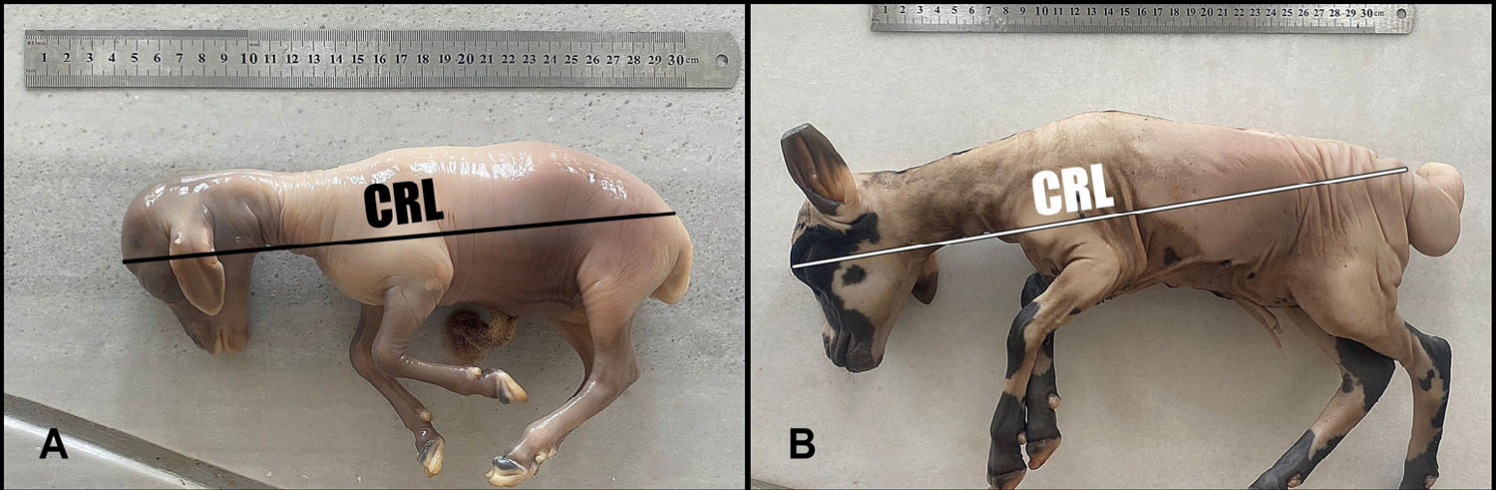
Crown‐rump parameter measurement. (A) Second trimester foetus and (B) third trimester foetus.

This study was carried out with 14 pregnant uteruses obtained from healthy cross‐bred Hamdani sheep (*Ovis aries*). Single pregnancies in pregnant uteruses were included in the study, while twin pregnancies were excluded. Pregnant uteruses were collected from healthy sheep slaughtered in the local slaughterhouse in Siirt province. Foetuses were removed from the collected uteruses, and amniotic fluid was cleaned. In order to accurately determine the gestational age of the collected uteruses, the foetal age was calculated from certain measurements (Figure 1). In this process, the crown‐anus of the foetuses was measured meticulously, and then the day of pregnancy was calculated using the formula *X* = 2.1 (*Y*+17) (*X* = pregnancy in days, *Y* = crown‐anus length) (Işbilir and Güzel 2024; Güzel and Işbilir 2024; Noakes, Parkinson, and Gary 2001). The uteruses were divided into two groups: second trimester (mid‐termpregnancy, 50–100th days) and third trimester (latepregnancy, 100–150th days). In each group, seven uteruses were included in the study.

### 2.2 Macroscopic Examination and Morphometric Measurements

In uterine tissues at different stages of pregnancy, placentome structures were exposed by dissection after the removal of amniotic fluid. Placentome placement, shapes, and numbers were determined. All placentomes were counted manually. For each uterus, measurements were taken from a minimum of 30 and a maximum of 95 placentomes. Morphometric measurements were carried out as placentome lengths, widths, and depths. Images were transferred to Image J (1.4), and placentome length and width parameters were measured using this program. A ruler was placed next to each uterine tissue as a scale for the program. Placentome depths were measured manually using a digital caliper (Mitutoyo, CDN‐20C, Japan).

### 2.3 Histological Examinations and Histomorphometric Measurements

Placentomes were fixed in %10 formaldehyde (pH 6.9–7.1) for 24 h at room temperature. Tissue samples were embedded in the paraffin after routine histological processing. The paraffin blocks obtained were cut with a microtome at a thickness of 5 µm. For the histological examination and the histometric measurements, the sections were stained according to the trichrome staining method of Crossmon (1937). Placentome tissue sections were examined under a light microscope (DM750; Leica) integrated with a digital camera (MC170; Leica).

Using a 20X objective, seven randomly selected areas (including the base, villus apex, and maternal crypts) were photographed from each prepared tissue section. According to a study by Diaz et al. (2015), caruncular and cotyledonary areas, capillary vessel numbers, and areas were measured from these photographs using the Qupath v0.5.0 software. In the study, both the number and area of caruncular capillaries within each caruncular area, as well as the area and number of cotyledonary capillaries within each cotyledonary area, were measured as described earlier (Diaz et al. 2015).

### 2.4 Statistical Analysis

The Minitab software (version 21.4.1) was utilized for all statistical analyses. Descriptive statistics were employed to summarize the distribution of various parameters including the number of placentomes, placentome length, width, depth, and pregnancy days. Measures of central tendency and variability were reported using Mean ± SE values along with median, minimum, and maximum values. To compare morphometric and histometric placentome measurements between the second and third trimesters of pregnancy, an independent sample *t*‐test was conducted. Statistical significance was set at *p* < 0.05.

## 3 Results

### 3.1 Macroscopic and Morphometric Findings

**FIGURE 2:**
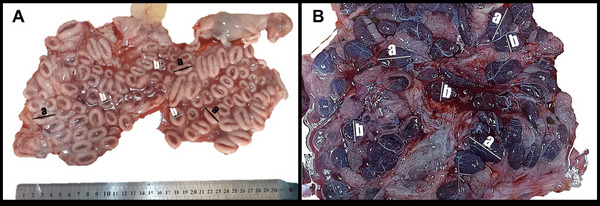
Second and third trimester uterus and placentome (A) and second trimester foetus and pregnant uterus. (B) Third trimester uterus. a: placentome length; b: placentome width.

The placentome structure consisted of a basal plate of maternal caruncular tissue and a chorionic plate of foetal cotyledonary tissue. It was determined that the placentome structure creates a strong bond between the mother and the offspring. Placentomes, which were observed as thick‐sided concave structures in the second trimester of pregnancy, had an appearance similar to the second trimester at the beginning of the third trimester (Figure 2A). In the period close to delivery (late third trimester), they were identified as thin‐edged structures with a slight depression in the centre. At the end of the third trimester, inverted structures were observed between these thin‐sided placentomal structures, with the caruncular layers on the inside and the cotyledon layers on the outside (Figure 2B). Placentomes were randomly distributed in the second and third trimesters without any particular order or sequence. In terms of size, the placentome size reached a maximum at the placental level (umbilical cord) and decreased at the horn tips. Small placentomes were identified among the randomly distributed placentomes.

Table 1 shows descriptive statistics of measurements of placentomes and pregnancy days in the second and third trimesters of pregnancy. The number, width, depth, and length of the placentome in the second and third trimesters are presented in Table 2. The length, width, and depth of the placentome in the third trimester were higher compared to the second trimester (*p* < 0.001). However, there was no difference in the number of placentomes in the second and third trimester (*p* > 0.05) (Table 2).

**TABLE 1:** Descriptive statistics of measurements of placentomes and pregnancy days in the second and third trimesters of pregnancy.

	Mean ± SE	SD	Minimum	Median	Maximum
Second trimester (*n* = 7)					
Number of placentomes	95.80 ± 4.40	10.72	85	94.5	113
Placentome length (cm)	2.27 ± 0.04	0.87	0.73	2.17	5.71
Placentome width (cm)	1.61 ± 0.02	0.58	0.50	1.51	3.79
Placentome depth (cm)	0.98 ± 0.02	0.31	0.36	0.96	1.84
Pregnancy days	82.67 ± 3.05	7.47	72	84	92
Third trimester (*n* = 7)					
Number of placentomes	98.30 ± 8.40	20.51	75	98	128
Placentome length (cm)	2.72 ± 0.04	0.95	0.97	2.58	7.52
Placentome width (cm)	2.02 ± 0.03	0.67	0.48	1.94	5.01
Placentome depth (cm)	1.14 ± 0.02	0.27	0.49	1.13	1.81
Pregnancy days	122.83 ± 2.76	6.77	112	124.50	130.00

Abbreviations: SD, standard deviation; SE, standard error of the mean.

**TABLE 2:** Comparison of placentome measurements between the second and third trimesters of pregnancy.

	Second trimester	Third trimester	*p* value
Number of placentomes	95.80 ± 4.40	98.30 ± 8.40	0.536NS
Placentome length (cm)	2.27 ± 0.04	2.72 ± 0.04	0.0001^***^
Placentome width (cm)	1.61 ± 0.02	2.02 ± 0.03	0.0001^***^
Placentome depth (cm)	0.98 ± 0.02	1.14 ± 0.02	0.0001^***^

Abbreviation: NS, non‐significant.

*** *p* < 0.001.

### 3.2 Histological Findings

**FIGURE 3:**
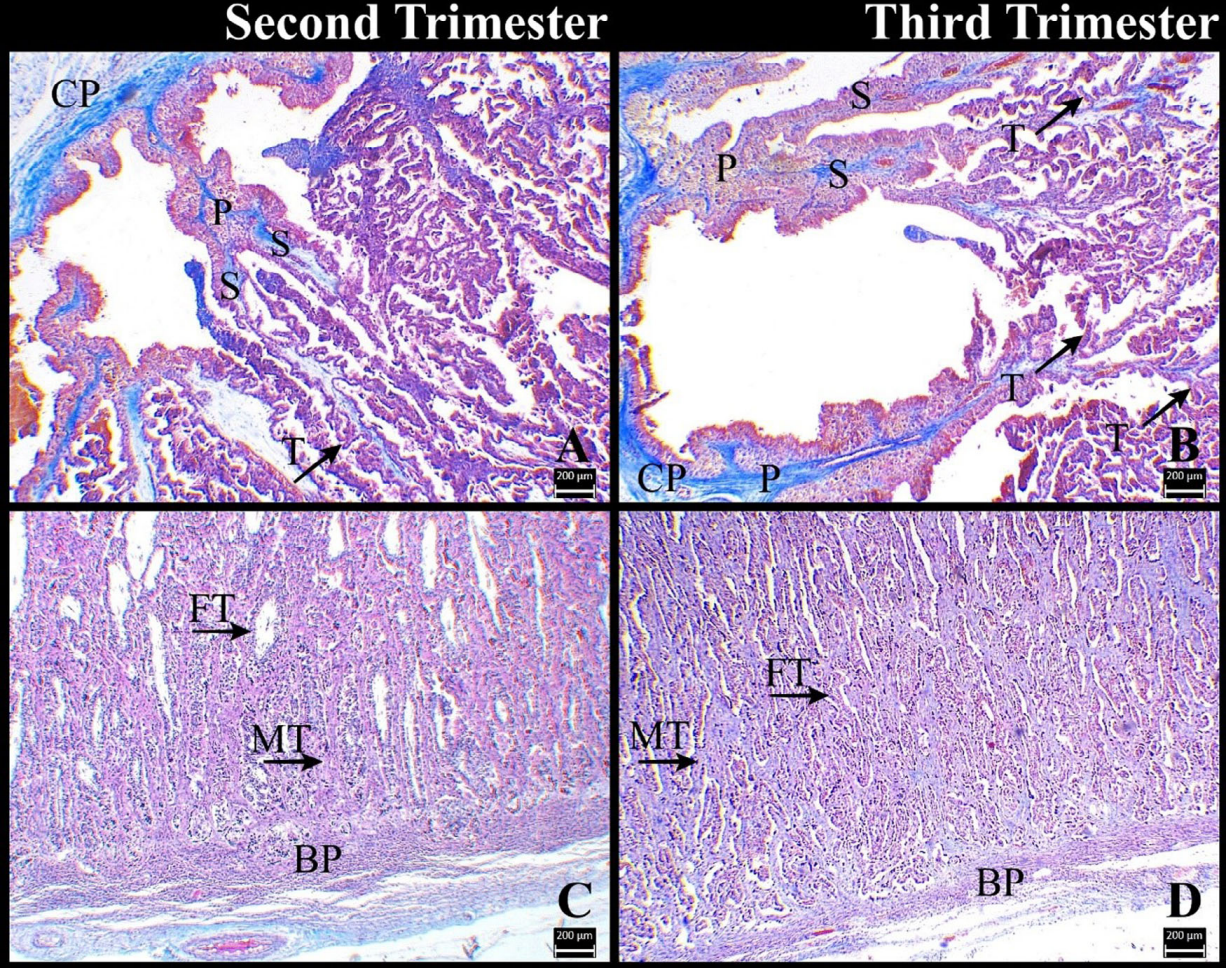
Chorionic villi and caruncular crypts. BP: basal plate; CP: chorionic plate; FT: foetal tissue; MT: maternal tissue; P: primary chorionic villi; S: secondary chorionic villi; T: tertiary chorionic villi. Crossmon's trichrome staining. Bar: 200 µm.

In the second and third trimesters of pregnancy, the foetal cotyledonary tissues formed from the chorionic plate of the placentome intertwined with the maternal caruncular tissues formed from the basal plate of the placentome (Figure 3). It was determined that several primary villi, separate from the chorionic plate of the placentome, extended towards the basal plate of the placentome. The primary chorionic villi emerged approximately at right angles from the chorionic plate and were oriented vertically to the basal plate. Several secondary chorionic villi were formed from the primary villi, either at an acute angle or at a right angle. Tertiary villi were formed by branching of secondary villi with smaller and more lateral branches. The resulting chorionic villus tree spread throughout the placentome (Figure 3A, B). More tertiary villi separated from secondary villi in the third trimester (Figure 3B). Similarly, the branching of caruncular crypts also increased in the third trimester. Thus, it was determined that maternal and foetal tissues were thoroughly intertwined in the third trimester (Figure 3D).

**FIGURE 4:**
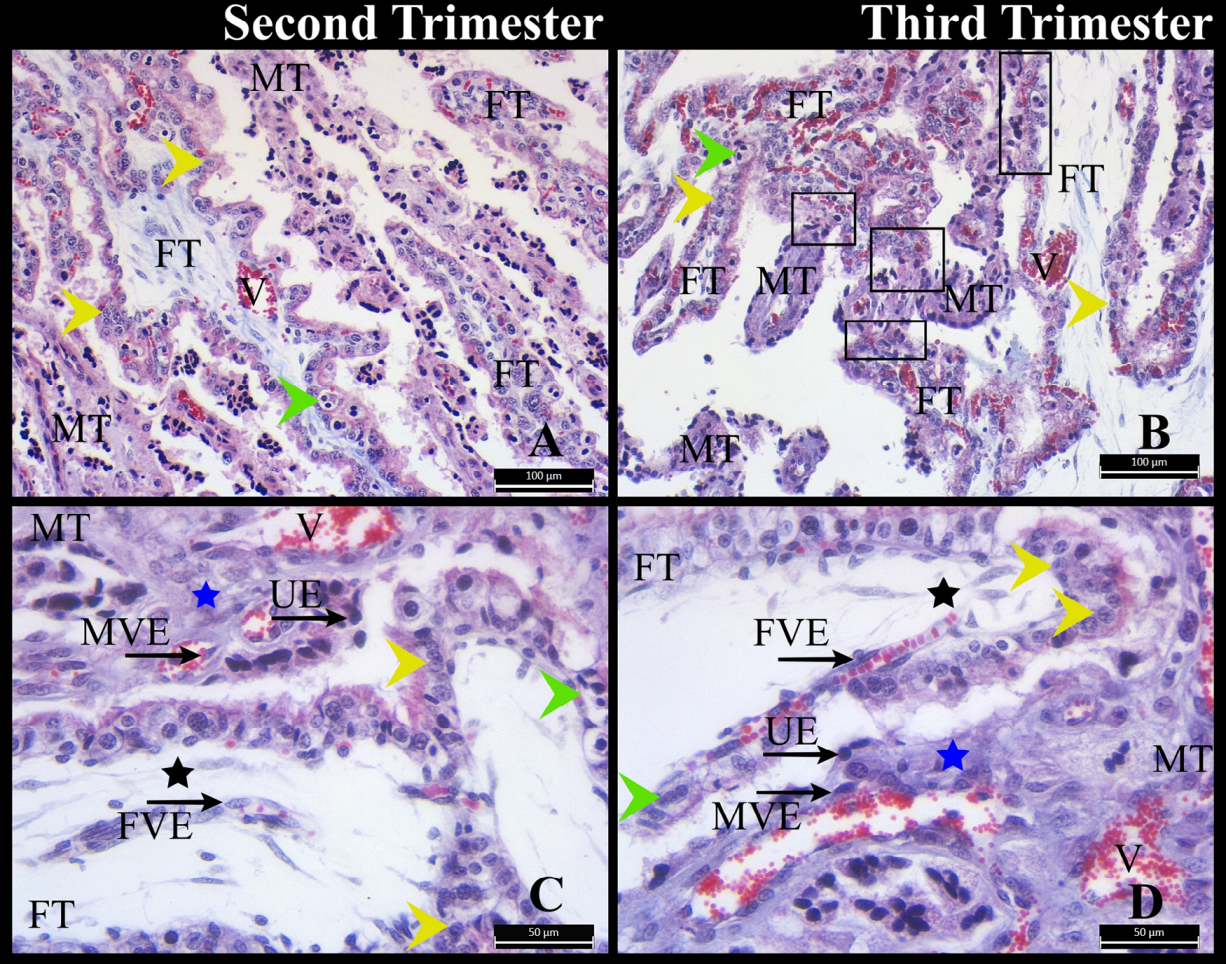
Chorionic villi and caruncular crypts. FT: foetal tissue; FVE: foetal vascular endothelium; MT: maternal tissue; MVE: maternal vascular endothelium; UE: uterine epithelium; V: blood vessel; yellow arrowhead: mononucleate trophoblast cell; green arrowhead: binucleate trophoblast cell; blue star: maternal connective tissue; black star: foetal mesenchyme; frame: intertwined of foetal and maternal tissue. Crossmon's trichrome staining. A and B: bar: 100 µm; C and D: bar: 50 µm.

The foetal villi were surrounded by maternal tissue (Figure 4A–D). The maternal–foetal connection was composed of foetal vascular endothelium, foetal mesenchyme, foetal trophoblastic epithelium, maternal uterine epithelium, maternal connective tissue, and maternal vascular endothelial layers (Figure 4C,D). It was seen that foetal villi consist of a vascularized mesenchyme with a simple layer of trophoblastic epithelial cells. Mononucleate trophoblast cells and binucleate trophoblast cells were observed to be randomly distributed in the trophoblastic epithelium. In both periods, mononucleate trophoblast cells were predominant in the foetal trophoblastic epithelium. Maternal crypts consisted of vascularised connective tissue and a single layer of cuboidal or columnar epithelium (Figure 4A–D). In the third trimester, clusters of mononucleate and binucleate cells were more intermingled with the maternal crypt epithelium in some areas of the placentome (Figure 2B).

**FIGURE 5:**
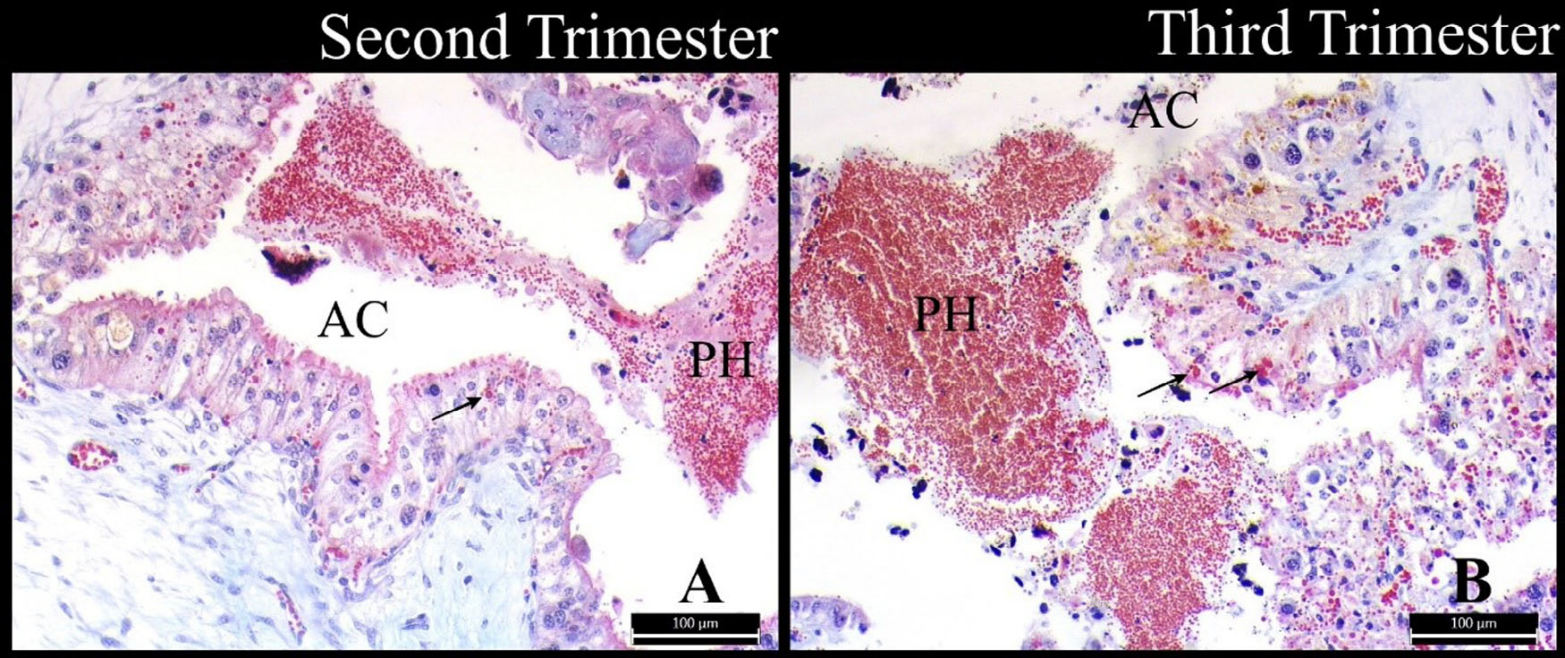
Placental hematomas in the arcade regions of the placentomes and erythrocytes in the cytoplasm of the trophoblast cells. AC: the arcade region; PH: placental hematomas; arrow: erythrocyte. Crossmon's triple staining. Bar: 100 µm.

In each period, placental hematomas were detected between the foetal and maternal tissues in the arcade regions of the placentomes, and erythrocytes were detected in the cytoplasm of the trophoblast cells (Figure 5). In the third trimester, it was noted that the erythrocytes observed in the cytoplasm of trophoblast cells were denser and that placental hematomas were larger (Figure 5B).

### 3.3 Histomorphometric Findings

The number and area of caruncular capillaries and cotyledonary capillaries measured histometrically in the second and third trimesters are presented in Table 3. Although there was no difference between the number of caruncular capillaries and the number of cotyledonary capillaries in the second trimester (*p* > 0.05), the number of cotyledonary capillaries was found to be higher than the number of caruncular capillaries in the third trimester (*p* < 0.001). In both periods, the caruncular capillary area was higher than the cotyledonary capillary area (second trimester for *p* < 0.05 and third trimester for *p* < 0.001). In the third trimester compared to the second trimester, there was no difference in the caruncular capillary number (*p* > 0.05), but the caruncular capillary area was higher (*p* < 0.001). However, both cotyledonary capillary number and area were higher in the third trimester compared to the second trimester (*p* < 0.05) (Table 3).

**TABLE 3:** Comparison of histometric measurements between the second and third trimesters of pregnancy.

	Second trimester	Third trimester	*p* value
Caruncular capillary number	11.48 ± 0.71	12.23 ± 0.64	0.428NS
Cotyledonary capillary number	11.95 ± 0.62	22.83 ± 1.1	0.0001^***^
*p* value	0.614NS	0.0001^***^	
Caruncular capillary area (µm^2^)	370 ± 20	580 ± 52	0.0001^***^
Cotyledonary capillary area (µm^2^)	296 ± 24	382 ± 25	0.014^*^
*p* value	0.020^*^	0.0001^***^	

Abbreviation: NS, non‐significant.

* *p* < 0.05.

*** *p* < 0.001.

## 4 Discussion

This study revealed macroscopic, morphometric, histological, and histomorphometric changes in placentomal tissues in the second and third trimesters of pregnancy in cross‐bred Hamdani sheep. In cattle, placentomes are reported to be organized very regularly in four rows along both uterine horns (Schlafer, Fisher, and Davies 2000). In this study, however, it was found that the placentomes were distributed randomly, without showing any order of arrangement, as in West African Dwarf goats (Igwebuike and Ezeasor 2013). In water buffaloes, Abd‐Elnaeim et al. (2003) reported that the shape of the placentome varies from circular to oval and quadrilateral; however, in general, it is mushroom‐shaped and has a rather short stalk on the maternal side. In Black Bengal goats (Khanam et al. 2016) and West African dwarf goats (Igwebuike and Ezeasor 2013), placentomes have been reported to be concave in shape. Kumar et al. (2015) reported that goat placentomes in late pregnancy were disc‐shaped, with shallow depressions and relatively thin edges. Similarly, in this study, placentomes were concave structures with thick edges in the second trimester and were thin‐sided structures with a slight depression in the centre in the third trimester. In addition, similar to the literature, placentomes were observed to have a short connection on the maternal side and their shapes were oval and circular in both trimesters.

In bovine, Laven and Peters (2001) reported that in late pregnancy, the number of placentomes did not increase in response to the foetus's growing nutritional demands. In West African Dwarf goats (Igwebuike and Ezeasor 2013) and yaks (Liu et al. 2010), it has been reported that the number of placentomes did not change with the development of the pregnancy. The number of placentomes did not change as the pregnancy progressed, as in West African Dwarf goats, bovine, and yaks. However, in goats, Kumar et al. (2015) reported that the number of placentomes varied from 92 to 153, which increased from early to mid‐pregnancy and decreased in late pregnancy. In addition, in sheep and goats, Kashoma and Luziga (2019) reported that the number of placentomes ranged from 70 to 92 and 74 to 104 in mid and late pregnancy, respectively. In our study, we determined the number of placentomes in Hamdani cross‐bred sheep as 95.80 ± 4.40 in the second trimester and 98.30 ± 8.40 in the third trimester. Although there are contrary findings in sheep and goats, our results suggest that the number of placentomes did not change between the second and third trimesters. Opposite findings on the number of placentomes between studies may be due to differences in the placentation process of the animal species and sample size. In addition, the size of placentomes ranged between 1.69 and 3.06 between days 45 and 140 of pregnancy in African dwarf goats (Igwebuike and Ezeasor 2013). Similarly, in goats, Kumar et al. (2015) determined that the length, width, and thickness of the placentomes increased as pregnancy progressed. In Balami and Yankasa sheep, it was found that the length and width of placentomes increased with the progression of pregnancy and placental thickness decreased only in late pregnancy (Gazali et al. 2023). In Yak, Liu et al. (2010) showed that there was a significant increase in the average size of the placentomes, but a slight decrease at 211 days. In the present study, the length, width, and depth of the placentomes in the cross‐bred Hamdani sheep increased as the progression of the pregnancy. The increase in placentome size as pregnancy progresses may be due to increasing nutritional requirements of the foetus during the late pregnancy.

As in sheep (Gazali et al. 2023; Majeed, Shalal, and Mohammed 2012) and goats (Igwebuike and Ezeasor 2013; Stephen et al. 2022), in the present study, it was found that cotyledonary and caruncular tissues were formed in the placentome and were intertwined with each other. It was determined that the chorionic villus tree had formed and spread throughout the entire placentome, as in goats (Igwebuike and Ezeasor 2013). In sheep, Hafez et al. (2010) stated that maternal crypt depth increases with foetal villi branching, and that foetal villi branching affects maternal crypt development. Researchers stated that this extensive branching probably allows greater invasion and surface contact with maternal tissues. Extensive branching might result from the site‐specific production of growth factors in foetal or maternal tissues (Hafez et al. 2010). Similarly, the branching of foetal villi and maternal crypts also increased in the third trimester in this study. As in Balami and Yankasa sheep (Gazali et al. 2023) and West African Dwarf goats (Igwebuike and Ezeasor 2012), mononucleate trophoblast cells and binucleate trophoblast cells were identified in the foetal trophoblastic epithelium. Also, as in sheep (Gazali et al. 2023), clusters of mononucleate and binucleate trophoblast cells wintermingled with the maternal crypt epithelium in some areas of the placentome as the pregnancy progressed.

The presence of placental hematomas in the feto‐maternal interface at the arcade zones of placentomes has been documented in sheep (Burton, Samuel, and Steven 1976), goats (Igwebuike and Ezeasor 2012; Santos et al. 1996), and buffalo (Pereira et al. 2001). Burton, Samuel, and Steven (1976) have indicated that these hematomas may be extravasated maternal blood leaking from capillaries at the foetal–maternal interface in the arcade zones of the placentomes. It has previously been reported that trophoblast epithelial cells phagocytose erythrocytes in sheep (Myagkaya and Schellens 1981). Erythrophagocytosis occurs at the arcade zones of the placentomes, and trophoblast epithelial cells engulf erythrocytes from extravasated blood pools at the feto‐maternal interface (Igwebuike and Ezeasor 2012; Myagkaya and Schellens 1981). The extravasation of maternal blood in the placentomes of sheep and other ruminants, as well as erythrophagocytosis performed by the trophoblastic epithelium, has been reported as a significant mechanism for providing iron to the foetus (Burton, Samuel, and Steven 1976; Myagkaya and Schellens 1981; Pereira et al. 2001; Santos et al. 1996). Similarly, in this study, the presence of hematomas in the arcade zones of the placentomes and the increased amount of erythrocytes in the cytoplasm of trophoblast cells in the third trimester may be associated with the growing iron demand of the developing foetus.

Angiogenesis is defined as the process by which new blood vessels are formed from existing vasculature (Demir et al. 2007). Vascular growth in placental tissues commences at an early stage of pregnancy and persists throughout gestation (Reynolds and Redmer 1995). In goats, Diaz et al. 2015) reported that a significant increase in capillary area density was observed in both caruncular and cotyledonary tissues on day 100 compared to day 50 of pregnancy. The researchers also found that the capillary area density of the caruncular tissue was greater than that of the cotyledonary tissue on days 50 and 100 of pregnancy. In sheep, Stegeman (1976) reported that the vascular density of cotyledonary tissue increases from day 40 to mid‐term pregnancy, and more slowly thereafter. An empirical model of angiogenesis in the maternal and foetal parts of the placentome during the last two thirds of pregnancy has been developed in sheep (Reynolds et al. 2005). In this model, caruncular capillary beds grow mainly by increasing capillary size, with only small increases in capillary number or surface density, resulting in increased capillary area density. In contrast, the capillary beds of the cotyledons grow primarily by branching. This leads to a large increase in the number of capillaries (Reynolds et al. 2005). As in sheep (Borowicz et al. 2007), our results demonstrated that from the second trimester to third trimester of pregnancy, capillary number more rapidly increases in foetal tissue than in maternal tissue. In addition, the number of cotyledonary capillaries increased, and the number of caruncular capillaries did not change as pregnancy progressed. However, caruncular capillary area increased in the third trimester compared to the second trimester which shows an increase in caruncular capillary beds without increase in the capillary number. The cotyledonary capillary area also increased in the third trimester compared to the second trimester. Overall findings imply that changes in the capillary number and area in the both caruncles and cotyledons may be related to the nutritional requirements of the developing foetus.

In summary, as a first report in cross‐bred Hamdani sheep, we revealed that placentome size, width, and depth increased in the third trimester compared to the second trimester of pregnancy. In addition, the caruncular capillary area increases without any significant increase in the number of caruncular capillary vessels in the third trimester compared to the second trimester of pregnancy. On the other hand, both the cotyledonary capillary area and number increased with the progression of the pregnancy. Overall results indicate that placentomes undergo significant changes as structural and physiological from the second to the third trimester of pregnancy to allow both nutritional and gas exchange between the foetus and the mother in sheep. Therefore, more comprehensive studies regarding the molecular basis of these structural and physiological changes need to be carried out.

## Author Contributions

B.K. conceived the idea, carried out experimental work, and prepared the original draft of the manuscript. A.O.T. conceived the idea; carried out experimental work, statistical analysis, and supervisorship of the study; and prepared the original draft of the manuscript. D.K. conceived the idea, carried out supervisorship of the study, and prepared the original draft of the manuscript. F.I. carried out experimental work and prepared the original draft of the manuscript. M.Z.A. and B.C.G. carried out experimental work.

## Ethics Statement

This study was approved by the Siirt University Local Ethics Committee for Animal Experiments (Decision No. 2024/04/15).

## Conflicts of Interest

The authors declare no conflicts of interest.

## Data Availability

The data for this study are available from the corresponding author on reasonable request.
